# Clinicopathological and prognostic significance of regulatory T cells in patients with non-small cell lung cancer: A systematic review with meta-analysis

**DOI:** 10.18632/oncotarget.9130

**Published:** 2016-04-28

**Authors:** Sha Zhao, Tao Jiang, Limin Zhang, Hui Yang, Xiaozhen Liu, Yijun Jia, Caicun Zhou

**Affiliations:** ^1^ Department of Medical Oncology, Shanghai Pulmonary Hospital, Tongji University School of Medicine, Tongji University Medical School Cancer Institute, Shanghai, China

**Keywords:** regulatory T cells, Foxp3^+^, non-small cell lung cancer, prognosis, systematic review

## Abstract

The prognostic and clinicopathological value of regulatory T cells (Tregs) infiltration in patients with non-small cell lung cancer (NSCLC) remains undetermined. A comprehensive literature search of electronic databases (up to December 2015) was conducted. Relationship between Tregs infiltration and clinicopathological features, recurrence-free survival (RFS) and overall survival (OS) was investigated by synthesizing the qualified data. A total of 1303 NSCLC patients from 11 studies were included. The pooled hazard ratio (HR) for survival showed that high Tregs infiltration had no effect on RFS (HR = 2.03, 95% CI: 0.61–3.44, *P* = 0.708) and OS (HR = 1.20, 95% CI: 0.58–1.62, *P* = 0.981). High FoxP3^+^ Tregs infiltration was significantly associated with poor OS in NSCLC (HR = 3.88, 95% CI: 2.45–5.40, *P* = 0.000). Test methods, ethnicity and types of specimens had no effect on predicting prognosis of Tregs infiltration. While high Tregs infiltration was significantly correlated with smoking status [odds ratios (ORs) = 1.54, 95% CI: 1.15–2.08; *P* = 0.004], none of other clinicopathological characteristics such as gender, histological type, lymph node metastasis status, tumor size, vascular invasion, lymphatic invasion and pleural invasion were associated with Tregs infiltration. The present study demonstrated that high FoxP3^+^ Tregs infiltration was significantly associated with poor prognosis in NSCLC and smoking status.

## INTRODUCTION

Lung cancer is the most common malignant tumor and the leading cause of cancer death worldwide, with 1.6 million new cases and 1.38 million deaths annually [1, 2]. Non-small cell lung cancer (NSCLC) accounts for 80–85% of all lung malignancies and the overall 5-year survival of patients with NSCLC remains approximately 15–20% [3, 4]. Although the driver mutations including epidermal growth factor receptor (EGFR) and anaplastic lymphoma kinase (ALK) have revolutionized the treatment and prognosis of NSCLC, it just focused on the cancer cell intrinsic properties [5, 6]. More and more recent studies have begun to investigate the prognostic and clinicopathological role of tumor microenvironment (TME) in NSCLC [7, 8]. In the past, it had been supposed that the tumorigenesis and failure of cancer immunotherapy was due to the insufficient activation of immune system. Recently, increasing evidence indicates that inhibitory function plays the crucial role in the TME [6, 9]. One of the most significant inhibitory components is the regulatory T cells (Tregs).

Tregs are one of the most important cells in the TME. Tregs-mediated suppression of tumor-associated antigens has been proposed as a potential mechanism to explain the failure of anti-cancer immunity and the expansion of Tregs was found to be an hindrance to successful tumor immunotherapy [10]. The clinicopatnological and prognostic valueof Tregs in patients with NSCLC have been a long-standing topic of debate. Several studies have suggested that tumor infiltrating Tregs could predict the clinical outcomes in patients with NSCLC. Some of them showed that high Tregs infiltration was associated with poor prognosis [11, 12], while others revealed that high Tregs infiltration was correlated with better prognosis [13–15]. Moreover, there are some studies reported no significant association between the presence of Tregs and patient survival [16]. Whether Tregs infiltration had the prognostic potential in patients with NSCLC remains controversial.

The X-linked gene forkhead box P3 (*FOXP3*), encoding the transcription factor Foxp3, serves as a lineage specification factor for the development and function of CD4+CD25+ Tregs [17, 18]. To date, Foxp3 is considered to be the most specific Tregs marker. Foxp3+ Tregs have been shown to the essential suppressors of the anti-tumor responses by interfering with the release of cytolytic granule by cytotoxic T lymphocytes (CTLs) [19]. High tumor infiltration of Foxp3+ Tregs is supposed to be correlated with the poor outcomes. Indeed, this relationship has been demonstrated in a number of publications [13–15, 20]. However, a recent study showed that high Foxp3+ Tregs infiltration was significantly associated with improved survival in patients with NSCLC [12]. Whether Foxp3+ Tregs infiltration had the prognostic value in patients with NSCLC also remains conflicting.

Although there has been a meta-analysis on this topic, they analyzed the prognostic value of Foxp3+ Tregs in different types of cancer and only two publications referred to NSCLC [21]. There have been more than nine papers published since this meta-analysis was conducted. Moreover, it did not perform the subgroup analysis on NSCLC and the relationship between Tregs infiltration and clinicopathological features in patients with NSCLC. For these reasons, we conducted this meta-analysis to derive a more precise estimation of the clinicopathological and prognostic significance of regulatory T cells in patients with NSCLC.

## RESULTS

### Study selection

**Figure 1:**
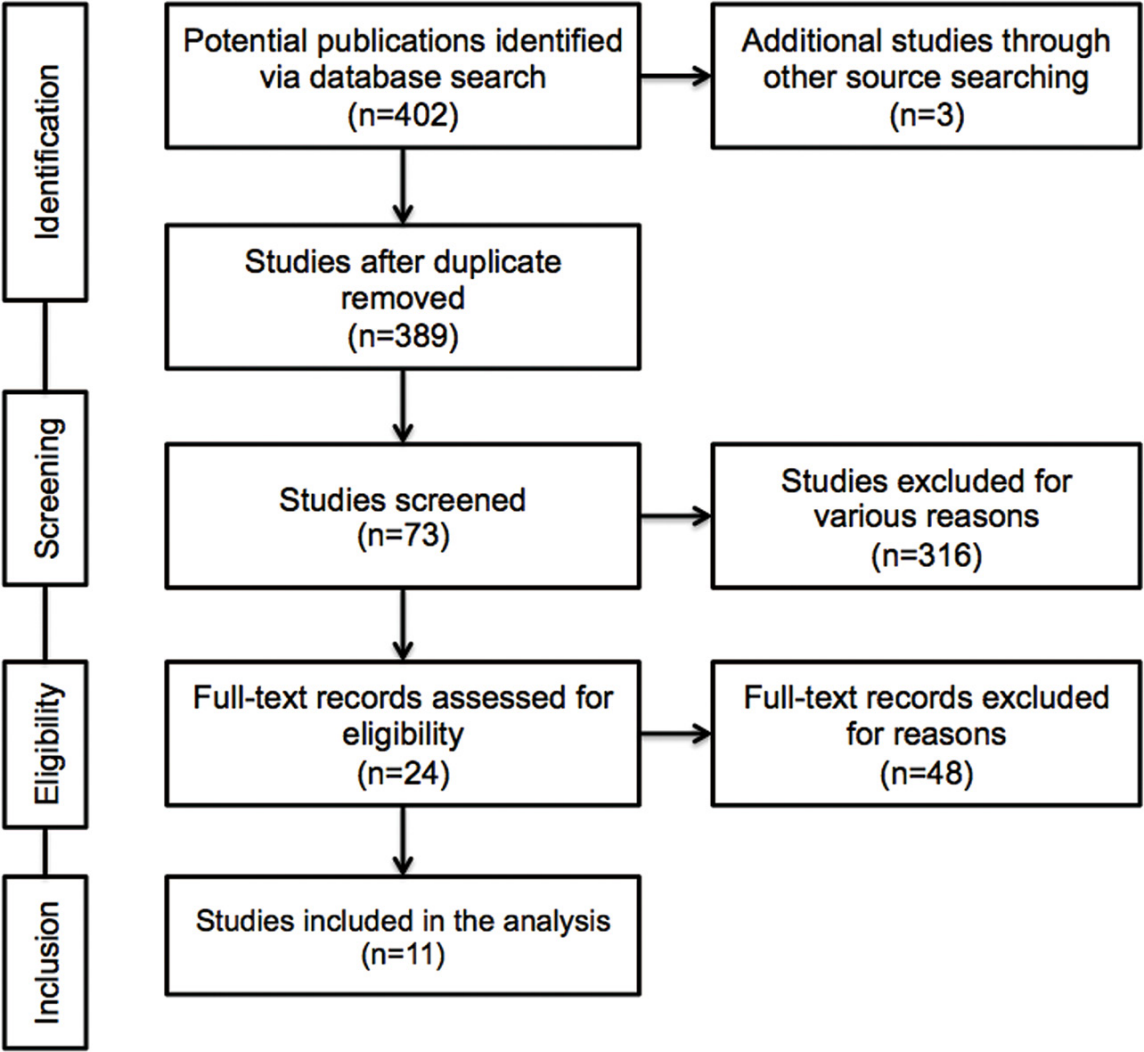
Flow diagram of the study selection process

The result of literature inclusion was showed in Figure 1. A total of 405 potentially relevant articles were found, and 11 studies were included in this analysis after screening [11–16, 20, 22–25]. Most of the excluded abstracts were reviews, letters or studies with insufficient data.

### Characteristics of the included studies

In the present analysis, a total of 1303 cases from 11 studies were met with our defined criteria and included. The main features of each eligible study were extracted (Table 1). Most of the studies (9/11) dealt with patients with stage from I to III/IV of NSCLC, and the rest of the studies (2/11) were focused on the patients with early stage I. Eight studies reported that surgery was performed on patients and two study mentioned that no prior treatment was applied, while only one had no relevant reports at all. The popular kinds of specimens included peripheral blood, regional lymph nodes and paraffin-embedded surgical tissues. The most commonly used test methods for Tregs infiltration are immunohistochemistry (IHC) and flow cytometry (FCM). Still, one study used the quantitative real time-polymerase chain reaction (qRT-PCR). Tregs markers refer to the different combinations of CD4^+^CD25^+^ and FoxP3^+^ or alone. One study had used the Helios+ as the specific marker of Tregs. The cutoff points of high Tregs infiltration were heterogeneous and only two studies used the median number of Tregs as the cutoff point. Eight included studies indicated RFS and/or OS and clinicopathological details. Among the eleven studies, three of them reported neither OS nor DFS, but presented clinicopathological features.

**Table 1:** General characteristics of included studies

Author	Year	Country	No. of Patients	Specimens	Test methods	Markers	Cut-off	Treg Positive Expression (%)	Stage	Pre-therapy	Outcomes
Liu et al.	2006	China	61	PB	FCM	CD4+ CD25+	< 3%	29.4	I–IV	Not applicable	Overall survival
Shimizu et al.	2010	Japan	100	Tumor tissues, RLN	IHC	Foxp3+	≥ 3 in 10 HPFs	51.0	I–III	Surgery	Recurrence-free survival
Erfani et al.	2012	Iran	23	PB	FCM	CD4+ CD25+ Foxp3+	Not applicable	Not applicable	II–IV	No prior treatment	Not applicable
Kayser et al.	2012	Germany	232	Tumor tissues	IHC	CD4+ CD25+	> median number	50.0	I–IV	Surgery	Overall survival
Tao et al.	2012	Japan	87	Tumor tissues	IHC	Foxp3+	≥ 25 in 10 HPFs	31.0	I–III	Surgery	Recurrence-free survival, overall survival
Hanagiri et al.	2013	Japan	158	PB, RLN	FCM	CD4+ CD25+ Foxp3+	> 0.5% of PBL, > 1.1% of RLNL	Not applicable	I–III	Surgery	Overall survival
Kinoshita et al.	2013	Japan	200	Tumor tissues	IHC	Foxp3+	≥ 6 in 1 HPFs	46.5	I	Surgery	Recurrence-free survival, overall survival
Hanagiri et al.	2014	Japan	131	Tumor tissues, PB, RLN	qRT-PCR	Foxp3+	2^−△CT^> 0.06	25.2	I	Surgery	Overall survival
He et al.	2015	China	50	PB	FCM	CD4+ CD25+	Not applicable	Not applicable	I–IV	No prior treatment	Overall survival
O’ Callaghan et al.	2015	Australia	197	Tumor tissues, RLN	IHC	Foxp3+	> median value*	43.9	I–IIIA	Surgery	Overall survival
Muto et al.	2015	Japan	64	Tumor tissues, PB	IHC, FCM	CD4+ Foxp3+ Helios-	> median number	50.0	I–IV	Surgery, chem therapy	Recurrence-free survival, overall survival

PB: peripheral blood; RLN: regional lymph nodes; FCM: flow cytometry; IHC: immunohistochemistry; HPFs: high-power fields; qRT-PCR: quantitative real time-polymerase chain reaction.

* median value was defined as the ratio of corresponding tumour islet and stroma counts.

### The prognostic effect of Tregs on survival

**Figure 2:**
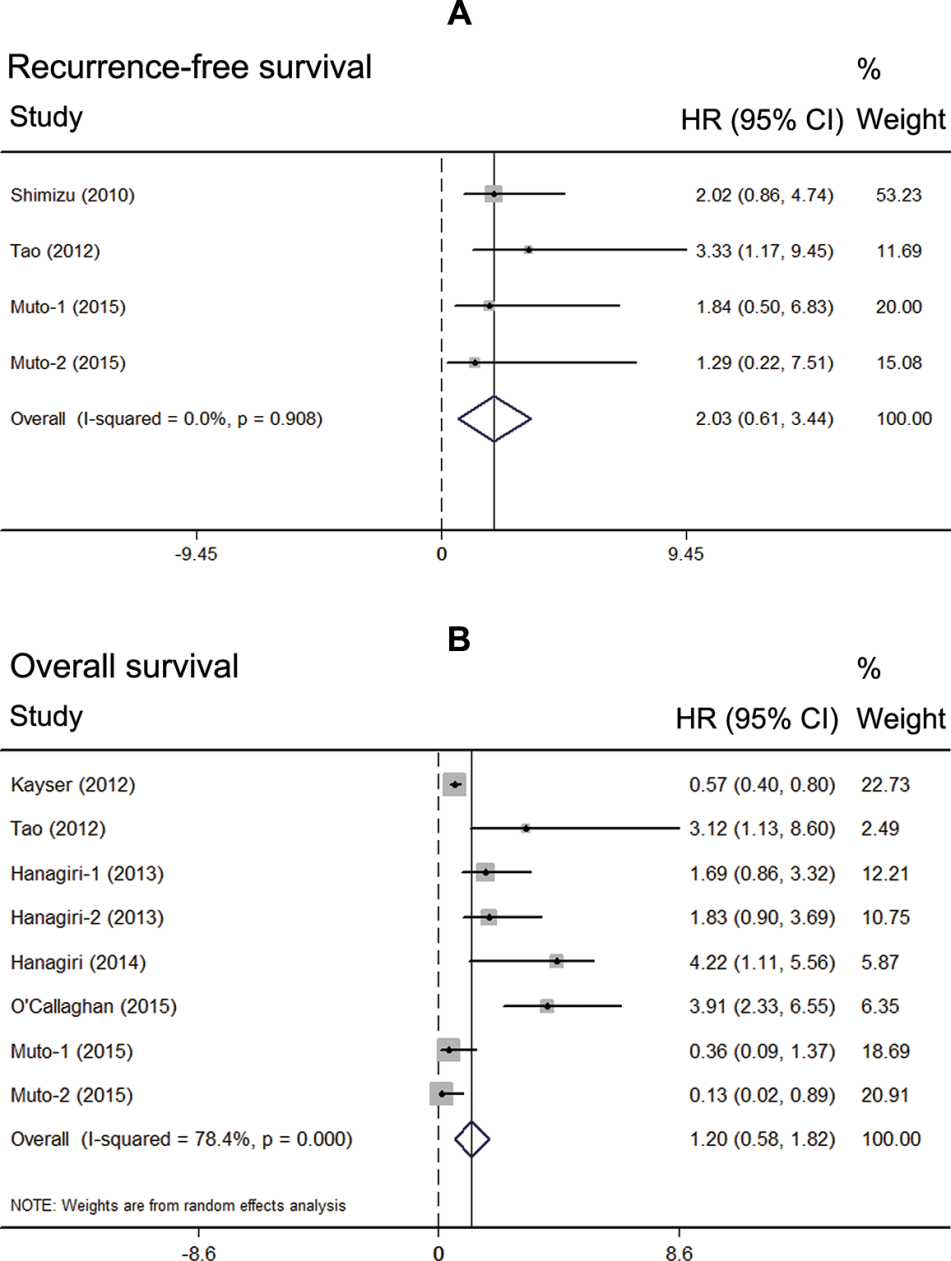
Prognostic value of high Tregs infiltration in NSCLC (**A**) meta-analysis of high Tregs infiltration and recurrence-free survival in NSCLC; (**B**) meta-analysis of high Tregs infiltration and overall survival in NSCLC.

Data of survival extracted from seven eligible studies were included in the meta-analysis. Since most of the included patients were with resection, patients with advanced stage in two studies were excluded when we performed the meta-analysis [22, 24]. Two of them reported the relationship between high Tregs infiltration and RFS, while six of them published the data of OS. Interestingly, meta-analysis found that the NSCLC patients with high Tregs infiltration showed no superior RFS than those with low Tregs infiltration (HR = 2.03, 95% CI: 0.61–3.44, *P* = 0.708, fixed model) (Figure 2A). Also patients with high Tregs infiltration showed no superior OS than those with low Tregs infiltration (HR = 1.20, 95% CI: 0.58–1.62, *P* = 0.981, random model) (Figure 2B).

### Subgroup analysis of the prognostic effect of Tregs

**Figure 3:**
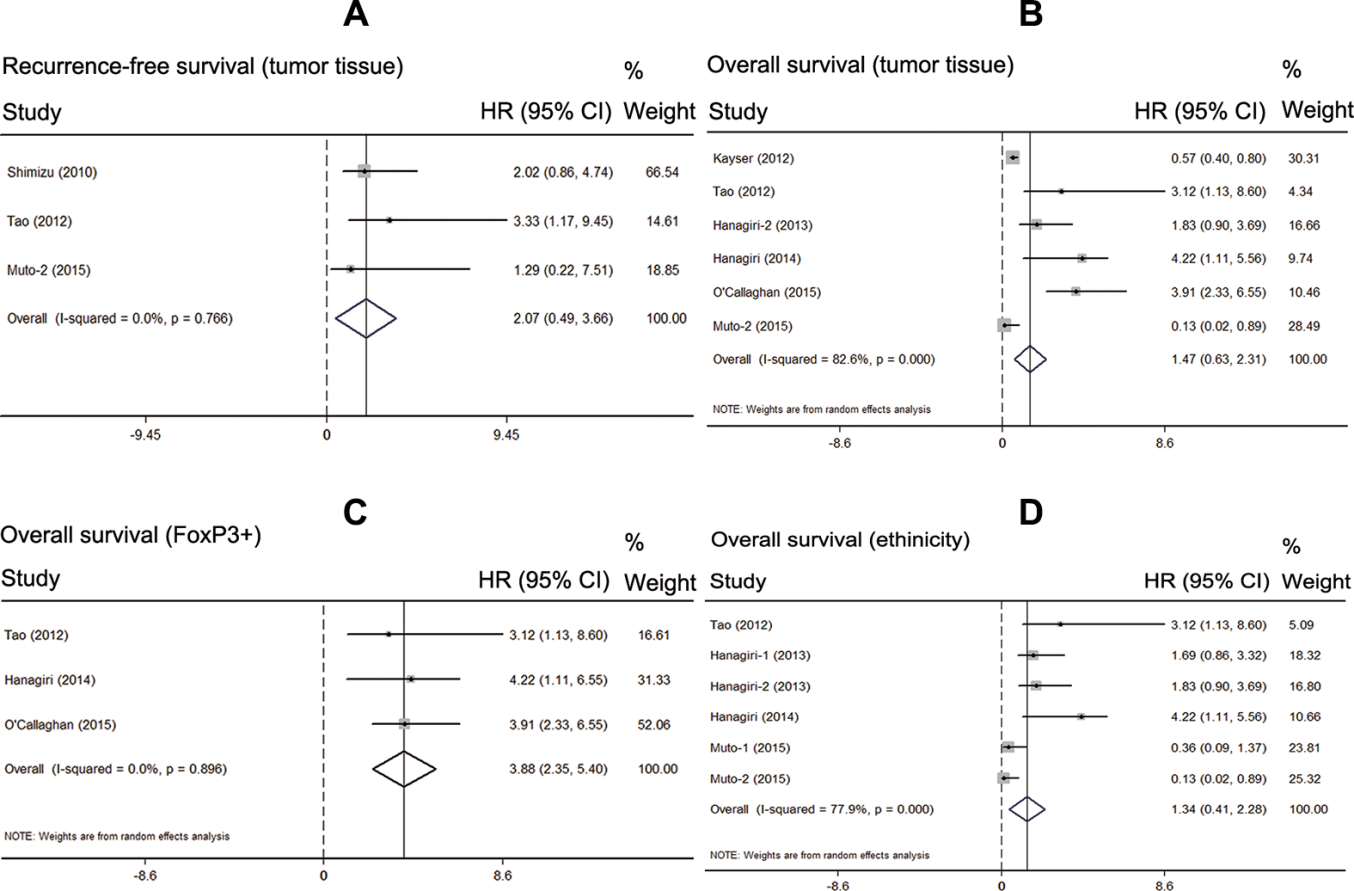
Subgroup analysis of the prognostic effect of Tregs (**A**) meta-analysis of high Tregs infiltration and recurrence-free survival in the studies used the tumor tissue; (**B**) meta-analysis of high Tregs infiltration and overall survival in the studies used the tumor tissue; (**C**) meta-analysis of high Tregs infiltration and overall survival in the studies used Foxp3+ as the Tregs marker; (**D**) meta-analysis of high Tregs infiltration and overall survival by same ethnicity.

Considering the high heterogeneity of pooled analysis on OS (I^2^ = 78.4%; *P* = 0.000), we performed the subgroup analysis of the prognostic effect of Tregs infiltration. Firstly, we analyzed the test method of Tregs infiltration and found no significant effect on OS (HR = 2.29, 95% CI: 0.33–4.90, *P* = 0.087). Then, we just included the studies used tumor tissues to test the Tregs infiltration. The pooled result showed that types of specimens had no significant effect on RFS and OS (*n* = 251, HR = 2.07, 95% CI: 0.49–3.66, *P* = 0.256; n = 869, HR = 1.47, 95% CI: 0.63–2.31, *P* = 0.681; respectively) (Figure 3A–3B). After that, we just included the studies used FoxP3^+^ as the markers of Tregs. The pooled result indicated that high FoxP3^+^ Tregs infiltration was associated with significantly poorer OS in patients with NSCLC (*n* = 415, HR = 3.88, 95% CI: 2.45–5.40, *P* = 0.000). In addition the results showed no obvious heterogeneity (I^2^ = 0.0%; *P* = 0.896) (Figure 3C). Finally, we investigated the ethnicity (Asian) of Tregs infiltration and found no significant effect on OS (*n* = 353, HR = 1.34, 95% CI: 0.41–2.28, *P* = 0.870).

### The relationship between Tregs infiltration and clinicopathological features

Four studies provided the information of various clinicopathological parameters and their correlation with Tregs infiltration was summarized in Table 2. Three studies had the statistical data on gender of patients. However, none of them reported the association between gender and high Tregs infiltration. Likewise, three studies demonstrated that there was no correlation between high Tregs infiltration and histology. In addition, Tao et al. showed that patients with smoking habits showed higher Tregs accumulation than non-smokers (*P* = 0.040). However, another two studies showed that there is no relationship between smoking history and FoxP3^+^ Tregs infiltration. Hence, we performed the meta-analysis and found that smoking status was significantly correlated with high FoxP3^+^ Tregs infiltration (OR = 1.54, 95% CI: 1.15–2.08; *P* = 0.004). Except for these above-mentioned parameters, controversies also existed on the correlation among tumor size (< 3 cm), lymph node metastasis, vascular invasion, lymphatic invasion and pleural invasion. The results of meta-analysis demonstrated that no correlation was found between the infiltration of FoxP3^+^ Tregs and tumor size (OR = 1.24, 95%CI: 0.89–1.74; *P* = 0.211), lymph node metastasis (OR = 0.98, 95%CI: 0.60–1.61; *P* = 0.944), vascular invasion (OR = 1.29, 95%CI: 0.80–2.09; *P* = 0.296), lymphatic invasion (OR = 1.05, 95% CI: 0.61–1.81; *P* = 0.848) and pleural invasion (OR = 1.49, 95% CI: 0.90–2.46; *P* = 0.119) (Table 2).

**Table 2:** Meta-analysis of the reported clinicopathological factors in the included studies

Factors	Number of Studies	Test of association			Test of heterogeneity		
		OR	95% CI	*p*	Q	I^2^	*p*
Gender (male)	4	0.99	0.79–1.25	0.938	0.45	0.0%	0.930
**Smoking**	2	1.54	1.15–2.08	0.004	0.12	0.0%	0.726
Adenocarcinoma	3	0.98	0.72–1.33	0.896	2.96	32.3%	0.228
Squamous cell carcinoma	3	1.36	0.82–2.52	0.235	0.64	0.0%	0.750
Lymph node metastasis	2	0.98	0.60–1.61	0.944	3.44	70.9%	0.064
Tumor size (< 3 cm)	2	1.24	0.89-1.74	0.211	1.54	35.2%	0.214
Vascular invasion	2	1.29	0.80–2.09	0.296	9.98	90.0%	0.002
Lymphatic invasion	2	1.05	0.61–1.81	0.848	4.93	79.7%	0.026
Pleural invasion	2	1.49	0.90–2.46	0.119	10.65	90.6%	0.001

RR: risk ratio; CI: confidence interval.

## DISCUSSION

Although a series of studies have discussed the prognostic value of Tregs in NSCLC, the results remains discordant. In the present article, the results indicated that when we ignored the different combinations of Tregs’ marker (including CD4+, CD25+ and Foxp3+), high Tregs infiltration had no effect on RFS and OS in NSCLC. However, when we just included studies used Foxp3+ alone or combined with CD4+ and CD25+ as the Tregs’ marker, we found that high Foxp3+ Tregs infiltration was significantly associated with OS in patients with NSCLC. Our results are consistent with these observations from other investigators. Previous studies have reported that NSCLC patients with high Foxp3+ Tregs infiltration were not only at increased risk of disease relapse but also correlated with unfavorable outcome [13–15, 26]. These studies confirm the prognostic value of Foxp3+ Tregs infiltration in patients with NSCLC.

Our study also found that high Foxp3+ Tregs infiltration was significantly associated with smoking status, and this correlation was in line with the studies that the increase in Tregs was related to smoking [13, 27, 28]. The mechanism of this phenomenon is not yet clear. One possible explanation revealed that cigarette smoking could promote the mobilization and accumulation of (MDSCs) and increase the level of interferon-γ in TME [29]. Meanwhile, other studies demonstrated that MDSCs could induce Foxp3+ Tregs development and expansion via the production of cytokines, such as IFN-γ, or direct cell-cell interactions [30–32]. Taken together, we can hypothesize that cigarette smoking can induce Foxp3+ Tregs infiltration via promoting accumulation of MDSCs. Nevertheless, this hypothesis need rigorously designed basic research to confirm.

Tregs are a heterogeneous population and consist of at least two subsets: natural Tregs (nTregs) and induced or adaptive Tregs (iTregs) [33]. More and more evidences show that two Tregs types play the different roles in the anti-tumor immune response [18, 34]. The nTregs originate in the thymus and are thought to recognize self-antigens [35]. The iTregs develop from conventional naïve T cell precursors at extra-thymic sites and are considered to suppress the immune response [36]. Therefore, clear discrimination between these two Tregs types is important for assessing their value in predicting prognosis. In the included study, Muto et al. used the Helios, a member of the *Ikaros* gene family, as a marker to distinguish iTregs from nTregs and found that Foxp3+Helios- Tregs could affect immune suppression, even in early stage NSCLC [12]. Moreover, their results suggested that patients with low level of Helios in Tregs had significantly poorer survival (HR = 0.13, *P* = 0.04). However, recent studies indicated that nTregs from iTregs are not distinguished only by Helios expression [37]. Other molecular markers should be given fully consideration including epigenetic Tregs-specific demethylated region (TSDR) modifications and neuropilin 1 [38–40]. Despite of these studies, a determined marker for nTregs has not been found so far.

Recently, several studies aimed to improve the therapeutic efficacy via depleting Tregs in patients with cancer. Some researchers attempted to inhibit Tregs function by using anti-CD25 antibody and by suppressing the cytotoxic T lymphocyte antigen 4 (CTLA4) pathway, which is an important inhibitory signal for activated T cells [41], but the results were disappointed. The reason may include that these strategies could not only deplete Tregs but also affect activated effector T cells function [42]. Of note, a recent study revealed that tumor-induced Tregs could proliferate via vascular endothelial growth factor (VEGF)/VEGF receptor (VEGFR) pathway in colorectal cancer [43]. VEGF/VEGFR pathway blockade did not eradicate all Tregs but simply restored their proportion to physiology levels. Therefore, it would be interesting to combine anti-angiogenic agents inhibiting VEGF/VEGFR pathway with other immunotherapeutic strategies [44, 45]. This approach would selectively suppress Tregs and avoid the depletion of effector T cells and minimize the occurrence of autoimmune mediated adverse effects correlated with a total of Treg depletion [43].

The present systematic review with meta-analysis has some limitations that should be acknowledged. Firstly, the number of the included studies was relatively small. Secondly, there may be some degree of publication bias in this area of research. We identified several abstracts describing articles that were not further detailed in standard publications. We have tried to contact authors of primary studies. However, we have not received any reply. Thirdly, there is clearly a multitude of confounding factors (test technique, specimen storage, laboratory condition and so on) that make experiment comparisons difficult. Last but not least, there is statistical heterogeneity among the studies regarding the prognostic value of Tregs infiltration. Studies may have differed with regard to the baseline characteristics of the patients included histological type, tumor size, disease stage, prior treatment and adjustments for other cofactors. Fortunately, we found that the heterogeneity may be due to the lack of standard test methods and evaluation criteria.

In conclusion, the current study suggests that high Foxp3+ Tregs infiltration may be a promising prognostic factor to patients with NSCLC. High Foxp3+ Tregs infiltration seems to correlate with smoking status instead of gender, histological type, lymph node metastasis status, tumor size, vascular invasion, lymphatic invasion and pleural invasion. Depletion of Tregs is an attractive strategy in future cancer treatment. In addition, the specific and exclusive surface molecule of Tregs should be illustrated clearly before large-scale clinical studies being performed.

## MATERIALS AND METHODS

### Publication search strategy

We performed a comprehensive publication search through the PubMed, EMBASE, Web of Science, and Cochrane library up to December 31, 2015, without language limitations. The following contextual query language was used: (“lung cancer”) AND (“regulatory T cells” OR “Foxp3”) AND (“prognosis” OR “mortality” OR “survival”). Titles and abstracts were reviewed to identify reports, which examined the association of Tregs expression with clinical outcomes, such as overall survival (OS), recurrence-free survival (RFS), disease free survival (DFS) and clinicopathological features. Reference lists of identified studies and reviews were also hand-searched. We have made any effort to contact authors of primary studies. This analysis was performed in accordance with Preferred Reporting Items for Systematic Reviews and Meta-Analyses: the PRISMA Statement [46].

### Inclusion and exclusion criteria

The criteria for inclusion were listed as follows: studies must have (1) been published as original articles; (2) evaluated human subjects; (3) Tregs was detected on tumor tissue or peripheral blood via testing the markers of CD4^+^CD25^+^, FoxP3^+^ or CD4^+^CD25^+^FoxP3^+^, rather than in the cell lines or any other kinds of specimens; (4) reported association of high and low Tregs infiltration with OS, DFS, or RFS; and (5) contained the minimum information necessary to estimate the effects (i.e., hazard ratio) and a corresponding measure of uncertainty (i.e., confidence interval, *P*-values, standard errors or variance). As an additional criterion, when a single population was reported in multiple reports, only the report with the most complete data was included to avoid duplication. Studies were excluded if they were: (1) reviews, case-only studies, or familial studies; (2) lacking sufficient data for calculation of incidence and/or HR with 95% confidence intervals (CIs); and (3) duplication of previous publications or replicated samples. Two reviewers determined study eligibility independently. Disagreements were solved by consensus.

### Data extraction

From each study, the following information was extracted: first author's name, year of publication, study population, Tregs infiltration assessment methods, cut-off definition, and incidence of high Tregs infiltration with 95% CIs, HR for OS, and/or DFS with corresponding 95% CIs. If the HRs and CIs were not reported, the total observed death events and the numbers of patients in each group were extracted to calculate HR and its variance indirectly. In order to guarantee the accuracy of collected data, studies for which only Kaplan-Meier curves would not be included. When both univariate analysis and multivariate analysis were reported to get the HR, the results of multivariate analysis were selected. Two reviewers extracted the data independently, using a predefined Excel form. Disagreements were resolved by consensus after discussion. If they can't get the consensus, the article would be excluded.

### Quality assessment

Two reviewers assessed the study quality independently by using the following factors: (1) distinct definition of the study population; (2) clear definition of the test method and the cut-off value of high Tregs infiltration; (3) sample size larger than ten and (4) clear definition of the outcome assessment (if applicable). Studies lacking any of these criteria were excluded.

### Statistical analysis

Overall survival (OS) was measured from the date of surgery until the date of death from any cause or the date on which the patient was last known to be alive. The recurrence-free survival (RFS) time was measured as the interval between the date of surgery and the date of recurrence, the date of death from any cause, or the most recent date on which the patient was last known to be disease-free. For time-to-event data, the HRs with their 95% CIs were directly extracted from the research article or calculated using previously published methods proposed by Tierney et al. [47]. The χ^2^ test was used to test for statistical heterogeneity and the I^2^ statistic was used to assess the extent of variability attributable to statistical heterogeneity across trials. *P* > 0.1 for the χ^2^ test and I^2^ < 25% were interpreted as signifying low-level heterogeneity. When there was no statistically significant heterogeneity, a pooled effect was calculated with a fixed-effects model; otherwise, a random-effects model was used.

To investigate the source of heterogeneity, predefined subgroup analyses were performed based on assessment method, cut-off definition, types of specimens and markers of Tregs. Sensitivity analyses were performed to assess the stability of the results, namely, a single study was deleted each time to reflect the influence of the individual data set on the results. Furthermore, we explored performed subgroup analysis on the relationship between high Tregs infiltration and clinicopathological parameters.

Statistical analysis was performed by STATA v12.0 (Stata Corporation, TX) and Review Manager 5.0 software. All data were analyzed using the Statistical Package for Social Sciences (SPSS) software (version 20.0 for Windows). *P* < 0.05 was considered statistically significant except for the *Q*-test.
